# Variable-Sensitivity Force Sensor Based on Structural Modification

**DOI:** 10.3390/s23042077

**Published:** 2023-02-12

**Authors:** Kazuto Takashima, Kengo Ota, Hiroki Cho

**Affiliations:** 1 Graduate School of Life Science and Systems Engineering, Kyushu Institute of Technology, 2-4 Hibikino, Wakamatsu-ku, Kitakyushu, Fukuoka 808-0196, Japan; 2 Faculty of Environmental Engineering, The University of Kitakyushu, 1-1 Hibikino, Wakamatsu-ku, Kitakyushu, Fukuoka 808-0135, Japan

**Keywords:** force sensor, shape-memory alloy, shape-memory polymer, cantilever, strain gauge, variable sensitivity

## Abstract

Force sensors are used in a wide variety of fields. They require different measurement ranges and sensitivities depending on the operating environment because there is generally a trade-off between measurement range and sensitivity. In this study, we developed a variable-sensitivity, variable-measurement-range force sensor that utilizes structural modification, namely changes in the distance between the force application point and the detection area, and changes in the cross-sectional area. The use of shape-memory materials allows the sensor structure to be easily changed and fixed by controlling the temperature. First, we describe the theory of the proposed sensor. Then, we present prototypes and the experimental methods used to verify the performance of the sensor. We fabricated the prototypes by attaching two strain gauges to two sides of a shape-memory alloy and shape-memory polymer plates. Experiments on the prototypes show that the relationship between the applied force and the detected strain can be changed by bending the plate. This allows the sensitivity and measurement range of the sensor to be changed.

## 1. Introduction

Force sensors (FSs) are applied not only in industry, but also in nursing and health care. For example, because the demand for continuous health monitoring has increased owing to an increase in health consciousness, the prevalence of infectious diseases, and an aging population, FSs are used to measure multiple biosignals, such as heart rate, the respiration cycle, and weight changes. Various FSs with different measurement ranges and sensitivities are required to match the measurement target in different operating environments because there is generally a trade-off between measurement range and sensitivity (MRS). For example, high-sensitivity, narrow-measurement-range FSs are used to measure small contact forces applied to soft fragile objects such as living tissues. On the other hand, in order to avoid signal saturation, low-sensitivity, wide-measurement-range FSs are used to measure large contact forces applied to rigid heavy objects in factories. Most FSs transform the mechanical deformation of the detection area under an applied force into a change in resistance, capacitance, or reflectance that can be measured using electric signals [1]. However, the MRS of a sensor cannot be changed after the sensor has been produced (see Section 2.1 for details).

Wide-measurement-range-sensitive FSs based on a quartz crystal resonator [2] and shape- and stiffness-memory ionogels [3] and a variable-configuration FS whose resolution can be adjusted via stiffness [4] have been proposed. Jiang et al. proposed a high-dynamic-range force/torque sensor that combines low- and high-strained bodies to detect force [5]. Okumura et al. proposed a high-dynamic-range force/torque sensor [6,7] that detects force in six axes and consists of multiple stages with different rigidities. Tamura et al. proposed a high-dynamic-range force/torque sensor that uses metal foil and semiconductor strain gauges (SGs) and a single stage [8]. Some FSs with a parallel-plate capacitive configuration [9,10] and based on microelectromechanical systems have been developed. Moreover, gain control methods for SGs have been proposed to maintain a wide dynamic range [11,12]. We previously developed an FS that uses a shape-memory polymer (SMP) that allows its MRS to be changed [13,14,15]. This sensor is unique in that it utilizes the stiffness change of the material caused by a temperature change.

SMPs [13,14,15,16,17,18], which are smart materials, change their modulus around the glass transition temperature (*T*_g_) and are often described as two-phase structures that comprise a lower-temperature “glassy” hard phase and a higher-temperature “rubbery” soft phase. The hard and soft phases are characterized by two different elastic modulus plateaus. The reversible change in the elastic modulus between the glassy and rubbery states of SMPs can be as high as several hundred-fold.

In previous studies [13,14,15], we made several prototypes of this sensor by attaching an SG to an SMP cantilever with an embedded electrical heating wire and evaluated their basic characteristics. Since the stiffness of an SMP depends on temperature, the measurable force range (determined based on the strain range) can be changed. Moreover, for a given strain resolution, the force resolution can be changed. In this way, the MRS of an FS can be changed by varying the temperature. Through experiments with these prototypes, which use the stiffness change of the SMP based on temperature, we showed that the MRS can be changed without replacing the actual sensor [13]. However, the changes in MRS (ranging from a hundred- to a thousand-fold) also depend on the Young’s modulus change of the SMP and are not adjustable. The Young’s modulus change may be too large for some applications. Therefore, we affixed a thin steel plate between the SG and the SMP in order to control the amount of stiffness change. Furthermore, we reduced the influence of the difference in the elastic modulus between the SGs and the SMP sheet. This made it possible to reduce the discrepancy between the theoretical and measured values. To describe the viscoelastic behavior more accurately, we proposed a transfer function using a generalized Maxwell model [14]. We verified the proposed model experimentally and estimated the parameters using system identification. In addition, we miniaturized the sensor and achieved the same performance as that in our previous study.

However, after the sensor is manufactured, the changes in MRS depend on the Young’s modulus change of the SMP around *T*_g_, and are not continuously adjustable. Therefore, in this study, we propose a variable-sensitivity FS that can change its structure. The concept of the proposed sensor can be applied in a wide range of fields because the proposed structural modification method is based on simple bending. The use of shape-memory materials (SMMs) allows the sensor structure to be easily changed and fixed by controlling the temperature. Since the MRS can be changed continuously, it is not necessary to replace the FS to match the measurement target. Numerous studies have developed an SMM sensor, including our previous research [13,14,15,17,19,20,21]. However, none of them developed a variable-sensitivity FS based on the structural modification of the SMM.

The rest of this article is organized as follows. In Section 2, we describe the theory of the proposed sensor. In Section 3, we present prototypes and the experimental methods used to verify the performance of the sensor. We design two sensors using an SMP or a shape-memory alloy (SMA) and evaluate these prototypes experimentally. In Section 4, we present and discuss the experimental results for the prototypes. Finally, in Section 5, we summarize the results.

## 2. Theory

### 2.1. Change in Distance between Force Application Point and Detection Area

**Figure 1:**
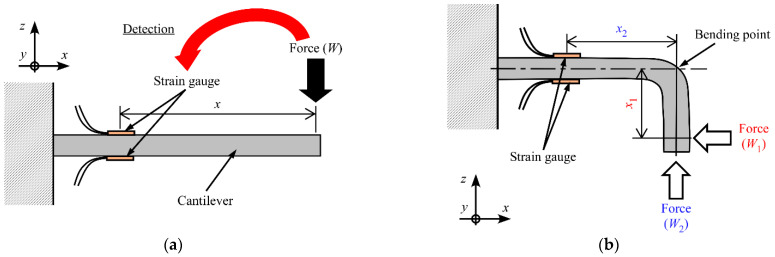
Bending state of beam. (**a**) Original shape. (**b**) Deformed and fixed shape.

First, we introduce a variable-sensitivity method that changes the distance between the force application point and the detection area. Some widely used FSs consist of SGs bonded to a bending beam, as shown in Figure 1a. Note that this design concept can be applied to FSs with different structures and measuring elements (i.e., it is not limited to SGs bonded to a cantilever beam). Under the assumption of an elastic cantilever, when a concentrated force (*W*) is applied along the −*z*-axis, the bending moment (*M*) can be expressed as follows [22]:

*M* = *EZε*, (1)

where *E* and *Z* are the elastic modulus and section modulus for the beam, respectively, and *ε* is the strain along the *x*-axis on the SG. The coordinate system is shown in Figure 1. In the variable-sensitivity method described here, because the absolute values of the strains on the two surfaces are the same, we can use a half-bridge system with two SGs. *M* can also be expressed as follows:

*M* = *Wx*, (2)

where *x* is the distance between the SG and the position at which the force is applied. From Equations (1) and (2), the relation between *W* and *ε* can be expressed as follows:

(3) $$W=\frac{EZ}{x}\varepsilon .$$

When the beam has uniform stiffness and the cross section of the beam is a rectangle, *Z* can be expressed as follows:

(4) $$Z=\frac{1}{6}bh^{2},$$

where *b* and *h* are the width and thickness of the beam, respectively. Substituting Equation (4) into Equation (3) yields the following equation for calculating *W*:

(5) $$W=\frac{bEh^{2}}{6x}\varepsilon .$$

Using Equation (5), we can calculate the applied force (*W*) from the strain measured (*ε*) by an SG attached to the beam (e.g., a stainless steel plate). However, since the deformation range depends on the sensor material and structure, the specifications cannot be changed after a sensor has been produced.

To overcome this limitation, in our previous studies [13,14,15], we developed an FS that uses an SMP sheet as the beam. As described in Section 1, since the *E* value for the SMP depends on temperature, the relationship between *ε* and *W*, namely Equation (5), can be changed. In this way, the MRS of the FS can be changed by varying the temperature. As an alternative method, Tamura et al. widened the measurement range by attaching two types of SG with different sensitivities on the surface of a cantilever beam [8].

In this study, we propose a variable-sensitivity FS, shown in Figure 1a, which when bent around the *y*-axis becomes fixed in the deformed shape shown in Figure 1b. The bending angle is 90°. When a load (*W*_1_) is applied to the tip along the −*x* direction, *M* can be expressed as follows:

*M* = *W*_1_*x*_1_, (6)

where *x*_1_ is the distance between the SG and the position at which *W*_1_ is applied. Therefore, from Equations (5) and (6), *W*_1_ can be calculated using the following equation:

(7) $$W_{1}=\frac{bEh^{2}}{6x_{1}}\varepsilon .$$

Similarly, when a load (*W*_2_) is applied to the tip along the +*z* direction, *W*_2_ can be calculated using the following equation:

(8) $$W_{2}=-\frac{bEh^{2}}{6x_{2}}\varepsilon ,$$

where *x*_2_ is the distance between the SG and the position at which *W*_2_ is applied.

Therefore, as shown in Equations (7) and (8), by changing the sensor structure, the relationship between the applied force (*W*_1_ or *W*_2_) and *ε* (consequently, the MRS) can be changed. For example, when *W*_2_ is measured, the effect of the force change on the strain becomes smaller because *x*_2_ (Figure 1b) is smaller than *x* (Figure 1a). That is, the measurement range of the FS becomes larger. A comparison between Equations (7) and (8) indicates that when *x*_1_ < *x*_2_, the measurement range for *W*_1_ is larger than that for *W*_2_ and the sensitivity to *W*_2_ is higher than that to *W*_1_. Note that because the bending position can be chosen arbitrarily, the MRS can be changed continuously. Moreover, as shown in Figure 1a, by bending the beam and fixing the bending shape, the detection direction of the applied force can be changed.

### 2.2. Change of Cross-Sectional Shape

**Figure 2:**
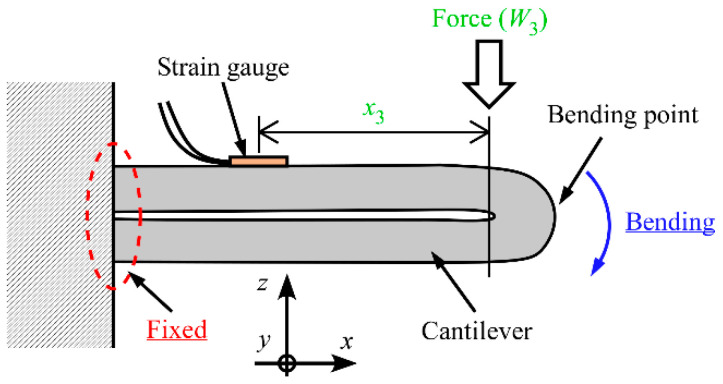
Bending state of beam. The cantilever is folded twice around the *y*-axis and the tip is fixed to the root of the beam.

The structural modification method described in Section 2.1 has some practical limitations, such as difficult mechanical assembly and the requirement of a large three-dimensional volume. Therefore, we next introduce a variable-sensitivity method that changes the cross-sectional shape of the sensor. In this method, because the strains on the two surfaces are different, we cannot use the half-bridge system with two SGs. As shown in Figure 2, the cantilever is folded twice around the *y*-axis and the tip is fixed to the root of the beam. By integrating the two-fold beam, the cross-sectional shape can be changed. Therefore, Equation (5) can be transformed into the following equation:

(9) $$W_{3}=\frac{2bEh^{2}}{3x_{3}}\varepsilon .$$

As described above, the relationship between the applied force (*W*_3_) and *ε* (consequently, the MRS) can also be changed.

**Figure 3:**
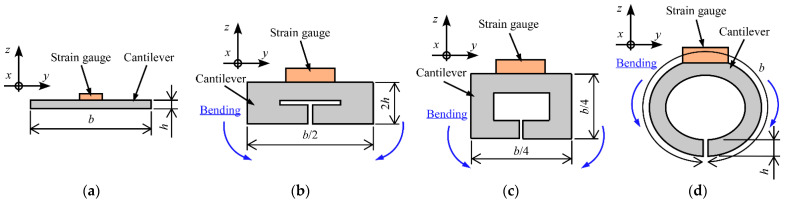
Modification of cross-sectional shape by bending around *x*-axis. (**a**) Original shape. (**b**) Thick rectangular plate. (**c**) Hollow rectangular column. (**d**) Hollow cylinder.

On the other hand, by bending the cantilever (Figure 3a) around the *x*-axis and fixing the bending shape, the relationship between the applied force and the strain can be changed. For example, when the cross-sectional shape is changed into a rectangular shape, as shown in Figure 3b, the width and thickness become 2*h* and *b*/2, respectively. Therefore, the section modulus (*Z*_1_) can be expressed as follows [22]:

(10) $$Z_{1}=\frac{\left(\frac{b}{2}\right){\left(2h\right)}^{2}}{6}=\frac{bh^{2}}{3}.$$

Moreover, as shown in Figure 3c, by bending the cantilever around the *x*-axis, a rectangular hollow column can be formed. Because the length of one side is *b*/4, the section modulus (*Z*_2_) can be expressed as follows [22]:

(11) $$Z_{2}=\frac{{\left(\frac{b}{4}\right)}^{4}-{\left(\frac{b}{4}-2h\right)}^{4}}{6\left(\frac{b}{4}\right)}=\frac{b^{4}-{\left(b-8h\right)}^{4}}{384b}.$$

Furthermore, as shown in Figure 3d, by bending the cantilever around the *x*-axis, a hollow cylindrical shape can also be formed. For a circumference of *b*, the outer and inner diameters are *b*/π and (*b*/π-2*h*), respectively. Therefore, the section modulus (*Z*_3_) can be expressed as follows [22]:

(12) $$Z_{3}=\frac{\pi }{32}\frac{{\left(\frac{b}{\pi }\right)}^{4}-{\left(\frac{b}{\pi }-2h\right)}^{4}}{\frac{b}{\pi }}=\frac{b^{4}-{\left(b-2\pi h\right)}^{4}}{32\pi^{2}b}.$$

As described above, by bending the cantilever around the *x*-axis and changing the section modulus, the MRS can be changed without changing the sensor length. Note that unlike for the method described in Section 2.1, it is not necessary to change the position and direction of the measured force.

**Figure 4:**
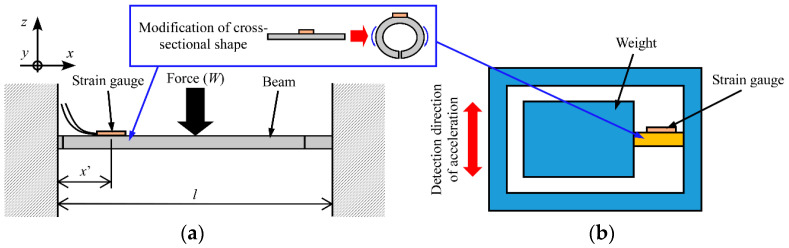
Applications of structural modification to (**a**) FS consisting of beam fixed at both ends and (**b**) accelerometer.

Alternative structures include a beam fixed at both ends, a beam supported at both ends, a curved beam, and a cross beam. In Figure 4a, both ends of the beam with an SG are fixed. When a concentrated force (*W*) along the −*z*-axis is applied at the center of the beam fixed at both ends, the bending moment (*M*) can be expressed as follows:

(13) $$M=\frac{W\left(l-4x^{\prime }\right)}{8},$$

where *l* is the distance between the two fixed ends and *x*′ is the distance between the position at which the force is applied and one fixed end. Using Equations (1) and (13), *W* can be expressed as follows:

(14) $$W=\frac{8EZ}{l-4x^{\prime }}\varepsilon .$$

As shown in Equation (14), by changing *Z*, the relationship between the strain (*ε*) and the applied force (*W*) can be changed. Therefore, this relationship can also be changed by bending the cantilever around the *x*-axis and changing the section modulus as shown in Figure 3.

This concept can be applied to other types of sensors, such as torque sensors and accelerometers (Figure 4b). For example, by changing the direction of the SG in Figure 1a, the torque applied to the cantilever can be measured. Because the relationship between the applied torque and the deformation also depends on the structure, a variable-sensitivity torque sensor can be obtained based on structural modification. Moreover, by changing the cross-sectional shape of a cross beam [6,7], it is possible to change the MRS of a six-axis FS.

Many velocity sensors and acceleration sensors measure the displacement of an object with respect to some reference object [1]. For example, as shown in Figure 4b, a weight is attached at the tip of an elastic body and the deformation of the elastic body is measured by an SG. When the sensor is accelerated, the elastic body deforms according to the inertia of the weight. The acceleration can be calculated from the deformation. In this configuration, similar to Figure 3, the MRS can be changed by changing the cross-sectional shape of the elastic body.

### 2.3. Application of SMM

The use of SMMs allows the sensor structure to be easily changed and fixed by controlling the temperature. Compared with SMAs, SMPs have the following advantages [17,18]:

1. Lower cost (1/25th that of SMA).
2. Lower density (1/7th that of SMA).
3. Larger recoverable strains (greater than 400% compared with a maximum of 8% for SMAs).
4. Easier creation of the complex shapes in Section 2.1 and Section 2.2 by heating (the change of the elastic modulus of SMP around *T*_g_ is large).

SMAs have the following advantages [17]:

1. Larger recovery stress (113 times that for SMPs).
2. Larger thermal conductivity (80 times that for SMPs).
3. Higher electrical conductivity.

For SMAs, austenite and martensite structures are stable at high and low temperatures, respectively. When an SMA is heated, it transforms from the martensite phase to the austenite phase and recovers its original shape. During the cooling process, it reverts to the martensite phase. During the heating cycle, the transformation starts and finishes when the austenite start temperature (*A*_s_) and the austenite finish temperature (*A*_f_) are reached, respectively.

## 3. Experiment

### 3.1. Prototype Sensors

In this study, we designed two sensors, one that used an SMP and one that used an SMA, and evaluated them experimentally. We bent and fixed the SMA and SMP plates as shown in Figure 1b.

#### 3.1.1. SMA FS

**Figure 5:**
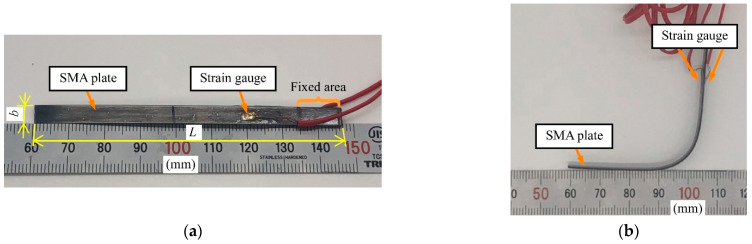

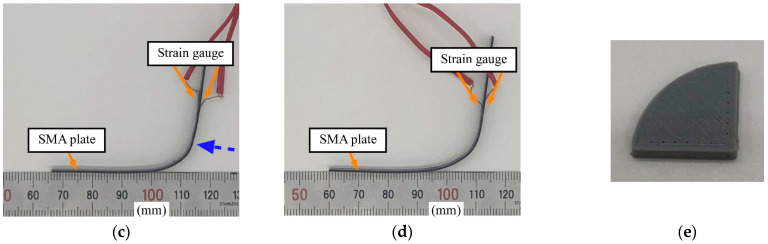
Prototype SMA FS. (**a**) Before bending. (**b**) Sample no. 1 (*x*_2_ = 23.8 mm). (**c**) Sample no. 2 (*x*_2_ = 21.1 mm). (**d**) Sample no. 3 (*x*_2_ = 18.9 mm). (**e**) Cylindrical jig used to bend SMA plates.

A photograph and the dimensions of the prototype SMA FS are shown in Figure 5 and Table 1, respectively. Note that the dimensions were not optimized; they can be scaled depending on the application. In this study, we prepared three samples with different *x*_1_ and *x*_2_ values, as shown in Figure 5b–d and Table 1. We bent the SMA plates and fixed the shape at room temperature. Using a cylindrical jig (Figure 5e) made by a 3D printer, the curvature radius of the bent part was set to 12 mm. Assuming an elastic beam, the maximum strain on the surface (*ε*_max_) can be expressed as follows [22]:

(15) $$\varepsilon_{\max}=\frac{h}{2R},$$

where *R* is the radius of curvature. Substituting *h* = 0.7 mm and *R* = 12 mm into this equation yields an *ε*_max_ value of 3%, which is smaller than the recoverable strain for an SMA (8% [17]).

In this study, we used Ti-Ni SMA plates. We heated the plates at 400 °C for 1 h. A straight shape was memorized. We measured the transition temperatures for the sample using differential scanning calorimetry (DSC). *A*_s_ and *A*_f_ were 34.6 and 53.7 °C, respectively. The SMA plates can be deformed and fixed at room temperature because the transition to the austenite structure and the recovery to the original shape do not occur. As shown in Figure 5, two SGs (KFGS-1-120-C1-16L1M2R, Kyowa Electronic Instruments Co., Ltd., Chofu, Japan) were attached at both sides of the SMA plate. We used a cyanoacrylate adhesive (CC-33A, Kyowa Electronic Instruments Co., Ltd.) to attach the SGs. With the half-bridge system, the SGs were connected to the bridge, one each to adjacent sides, with a fixed resistor inserted on the other sides.

#### 3.1.2. SMP FS

**Figure 6:**
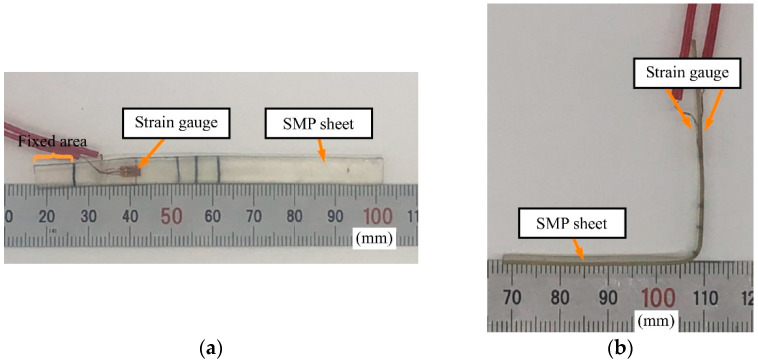
Prototype SMP FS. (**a**) Before bending. (**b**) After bending and fixing.

A photograph and the dimensions of the prototype SMP FS are shown in Figure 6 and Table 2, respectively. We chose a polyurethane SMP (MP4510, SMP Technologies Inc., Tokyo, Japan, *T*_g_ = 45 °C, elastic modulus: 1350 MPa below *T*_g_, 4.5 MPa above *T*_g_). We prepared an SMP sheet in a manner similar to that described in our previous studies [13,14,15]. Briefly, two liquid components were mixed, poured onto a plate, and cured. The thick nonuniform SMP sheet was then pressed and heated to make it uniformly thin via secondary shape formation. As was done for the SMA FS, we attached two SGs to the SMP FS.

We prepared one prototype sensor and changed the bending position after heating to above *T*_g_. SMPs can be deformed above *T*_g_ by applying a small load, and maintain their shape after they have been cooled below *T*_g_ (they are considered rigid in this state). In our previous study, we inserted a heating wire made of nichrome into the sheets to heat the SMP sheets and reduce their stiffness. However, for the FS proposed in this study, heating was necessary only when the shape was changed. Therefore, we did not insert a heating wire and instead heated the sample by blowing hot air onto it. The lack of a heating wire facilitates miniaturization and decreases the risk of failure. In a future study, we will incorporate a temperature control system into the jig used to bend the SMP. Such a system would require a large amount of power and would be bulky in order to shorten the heating and cooling times, and thus should be separate from the sensor from the viewpoint of miniaturization and cost.

In this study, *A*_s_ and *T*_g_ were different (34.6 and 45 °C, respectively). When these temperatures are close to room temperature, it is easy to heat the SMMs to change and fix the structure. However, the SMMs are strongly influenced by ambient heat. Therefore, it is necessary to tailor *A*_s_ and *T*_g_ according to the application and the purpose for which the FS will be used. Note that *T*_g_ can be set within a wide range (−40 to 120 °C).

### 3.2. Methods

**Figure 7:**
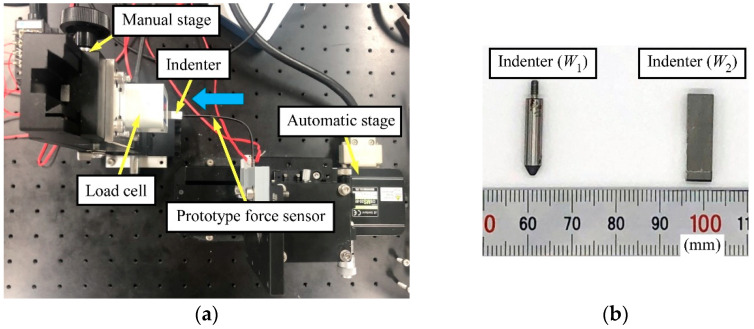
Appearance of (**a**) experimental apparatus (blue arrow shows sensor movement direction) and (**b**) indenters attached to load cell.

The experimental apparatus is shown in Figure 7a. The applied force and the strain were measured at room temperature. The relationship between the strain and the force applied using an indenter connected to the load cell was then evaluated. As shown in Figure 7b, according to the direction of the force, we used two types of indenter to maintain contact between the sensor tip and the indenter. The load cell (LVS-200GA, Kyowa Electronic Instruments Co., Ltd.) and the sensor were attached to a manual stage and an automatic stage (OSMS20-85, Sigma Koki Co., Ltd., Tokyo, Japan), respectively. The prototype sensor was automatically moved using the automatic stage. The distance between the fixed end and the SG on the prototype sensor was 15 mm. The SG was connected to a PC through a bridge box (DB-120A, Kyowa Electronic Instruments Co., Ltd.) and a strain amplifier (DPM-711B, DPM-912B, Kyowa Electronic Instruments Co., Ltd.). The load cell was connected to the PC through a strain amplifier. The sampling frequency was 100 Hz.

The sensor was deformed as follows:

Step 1: The sensor was held motionless in the unloaded state (just before touching).
Step 2: After the unloaded state, the sensor was moved in the direction of the blue arrow in Figure 7a and brought into contact with the load cell to apply a deformation of 1 mm to the tip of the sensor.
Step 3: With the tip deformed, the sensor was held motionless.
Step 4: The sensor was returned to the initial position.

Steps 1 through 4 were repeated twice. We set the velocity in Steps 2 and 4 to 0.5 mm/s and the rest time in Steps 1 and 3 to 10 s. For each condition, the measurements were conducted three times.

We compared the experimental results with the theoretical values calculated by substituting the measured sensor size (*b*, *h*, *x*_1_, and *x*_2_ shown in Table 1 and Table 2) and Young’s modulus into Equations (7) and (8). We used *E* = 34.5 GPa for SMA [17].

## 4. Results and Discussion

### 4.1. SMA FS

**Figure 8:**
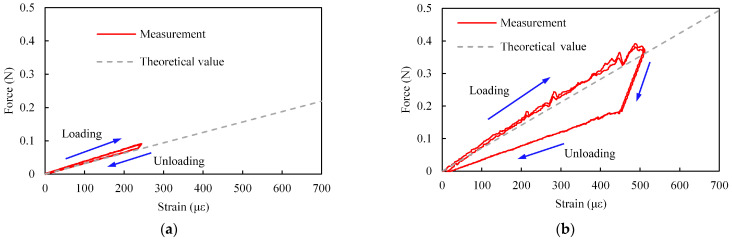
Example of relationship between applied force and strain (sample no. 2, *x*_2_ = 21.1 mm). (**a**) *W*_1_. (**b**) *W*_2_.

An example of the relationship between *W* and *ε* is shown in Figure 8. The theoretical values calculated from Equations (7) and (8) are also shown. In these figures, *W* and *ε* are the absolute values. In many cases, the measured values are similar to the theoretical values.

**Figure 9:**
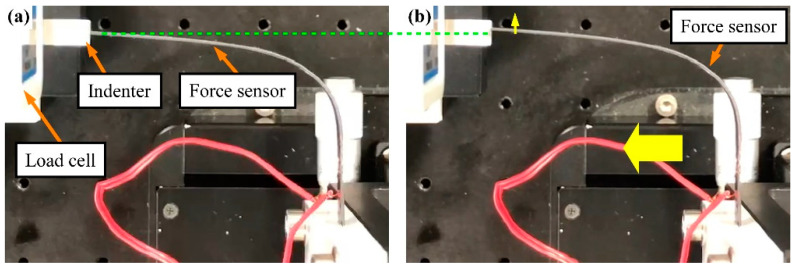
Movement of sensor tip (sample no. 2). (**a**) Before movement. (**b**) After 2-s movement of automatic stage. Yellow arrows show movement directions. The green dashed line shows the initial position of the sensor tip.

**Figure 10:**
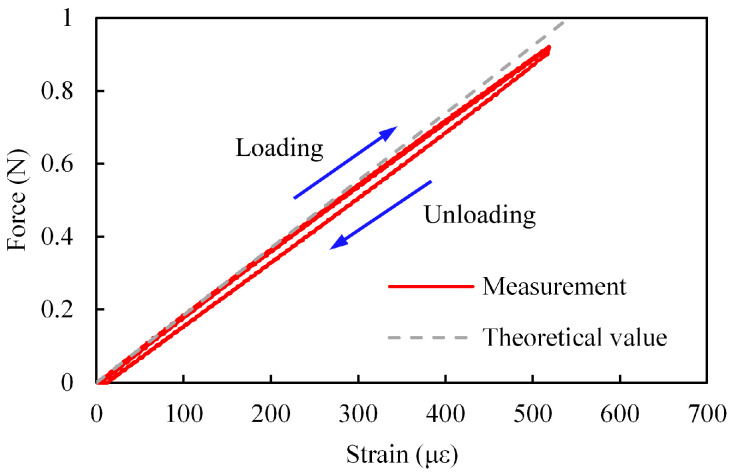
Example of relationship between applied force and strain (sample no. 2, *x* = 8.1 mm). A force was applied in the direction of the dashed arrow in Figure 5c.

As shown in Figure 8a, for all samples, the relationship between *W*_1_ and the strain was almost linear and the hysteresis was small. On the other hand, as shown in Figure 8b, when *W*_2_ was applied, there was a large hysteresis. One reason for the increase in hysteresis could be that the sensor tip moved on the indenter surface irregularly, as shown in Figure 9. Consequently, since *x*_2_ in Equation (8) and the force direction changed irregularly, the relationship between *ε* and *W* also changed. Moreover, the tip of the sensor buckled due to *W*_2_ and deformed. Since buckling is an unstable phenomenon, the relationship between *ε* and *W* could be easily changed by slightly changing the position and direction of the applied force. In order to evaluate the effect of buckling, we applied a force (*W*_2′_) in the direction of the dashed arrow in Figure 5c. An example of the relationship between *W*_2′_ and *ε* is shown in Figure 10. As shown, the relationship between *W*_2′_ and *ε* was almost linear and the hysteresis was small. Therefore, it may be useful to use *W*_2′_ instead of *W*_2_ to eliminate hysteresis.

In many cases, there is a difference between the measured force and the theoretical value. One reason for this difference could be that the Young’s modulus (*E*) and the dimensions used to calculate the theoretical values were not accurate. Another reason could be that the bending angle of the SMA plate was not exactly 90°, as shown in Figure 5 (the SMA plates were bent manually).

**Figure 11:**
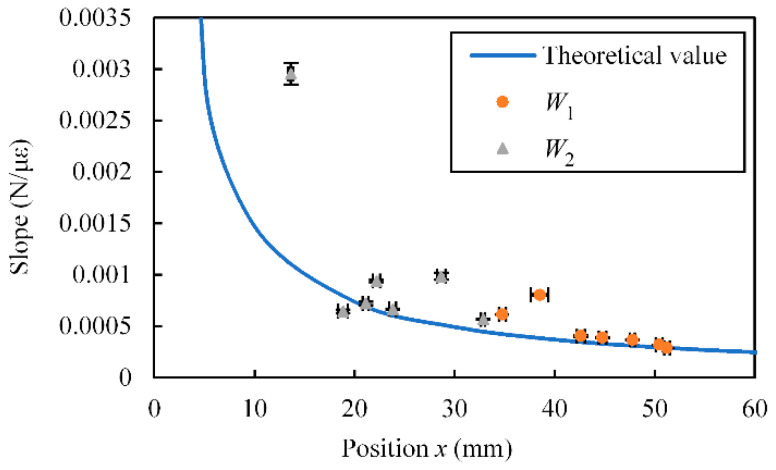
Relationship between *x* and slope for SMA FS.

From the relationship between the applied force and the strain obtained in the above experiment, we calculated the slope of the linear approximation formula. The relationship between the calculated slope (average ± standard deviation) and *x* is shown in Figure 11. The reciprocal of the slope (unit: με/N) corresponds to the sensitivity of the sensor. The theoretical values calculated using Equation (5) are also shown. As shown in this figure, similar to the case for the theoretical values, the slope changed according to *x*. The standard deviation was small.

### 4.2. SMP FS

**Figure 12:**
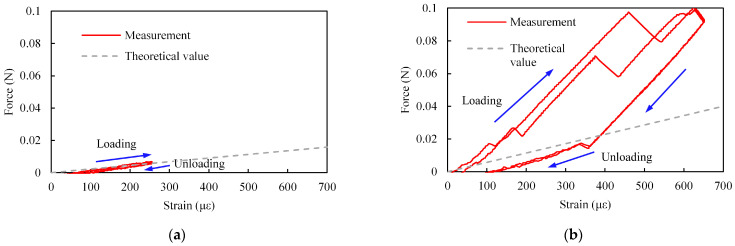
Example of relationship between applied force and strain for SMP FS (*x*_2_ = 17.8 mm). (**a**) *W*_1_. (**b**) *W*_2_.

**Figure 13:**
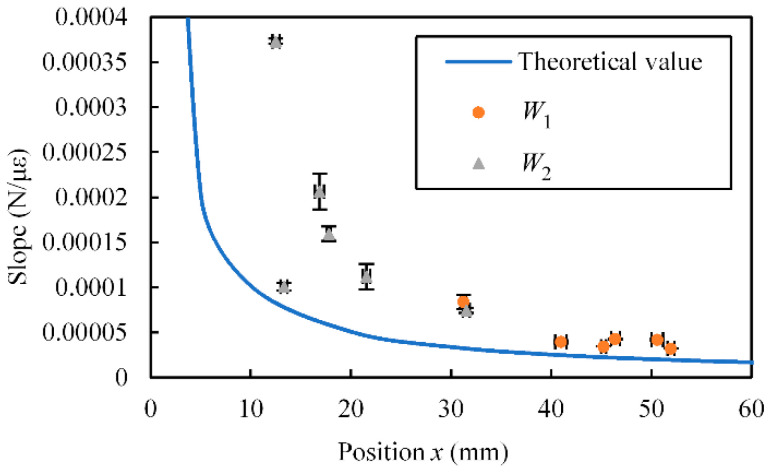
Relationship between *x* and slope for SMP FS.

An example of the relationship between *W* and *ε* for the SMP FS and the calculated slope are shown in Figure 12 and Figure 13, respectively. The theoretical values calculated from Equations (5), (7), and (8) are also shown in these figures. In these figures, *W* and *ε* are the absolute values. Similar to the case for the SMA FS, the relationship between the applied force and the strain can be changed by modifying the structure of the SMP sensor.

As shown in Figure 13, the slope for the SMP-based sensor was smaller than that for the SMA-based sensor even though the sensor sizes were similar. One reason for the difference in slope could be that the Young’s modulus for SMP is smaller than that for SMA. Since the Young’s modulus for the SMP is small, the measured force was small, as shown in Figure 8 and Figure 12. Therefore, the hysteresis and the variation in Figure 12b are larger than those in Figure 8b.

**Figure 14:**
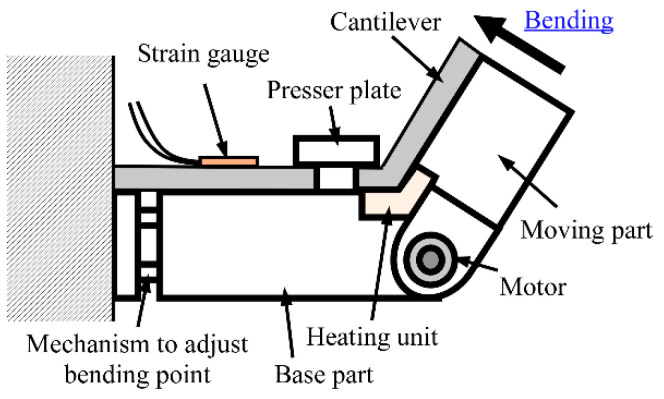
Jig used to bend and heat FS.

Similar to the case for the SMA FS, there was a difference between the experimental and theoretical values, as shown in Figure 13, which could be attributed to manufacturing errors. For example, since we manufactured and bent the sensor manually, the width and thickness were not constant, and the bending angle of the SMP plate was not exactly 90°. Moreover, the adhesion strength between the SMP and the SG and the small Young’s modulus of the SMP may have affected the SG measurements. In future studies, these errors will be minimized by using a jig and a manufacturing machine, and the structure of the sensor will be improved. For example, we will design the jig to bend and heat the sensor as shown in Figure 14.

The signal-to-noise ratio (*SNR*) of the proposed sensor is superior to that of a conventional fixed-sensitivity FS because the effect of electrical noise on the SG does not depend on sensor shape. In fact, in this study, the standard deviation of the measured strain was almost the same (0.66 με) in the unloaded state, despite the maximum applied force varying (0.07–0.83 N) due to the structural modification. Moreover, we calculated the *SNR* using the average (*m*) and the standard deviation (*σ*) of the slope in Figure 11 and Figure 13 as follows:

(16) $$SNR=20\log_{10}\frac{m}{\sigma }.$$

**Figure 15:**
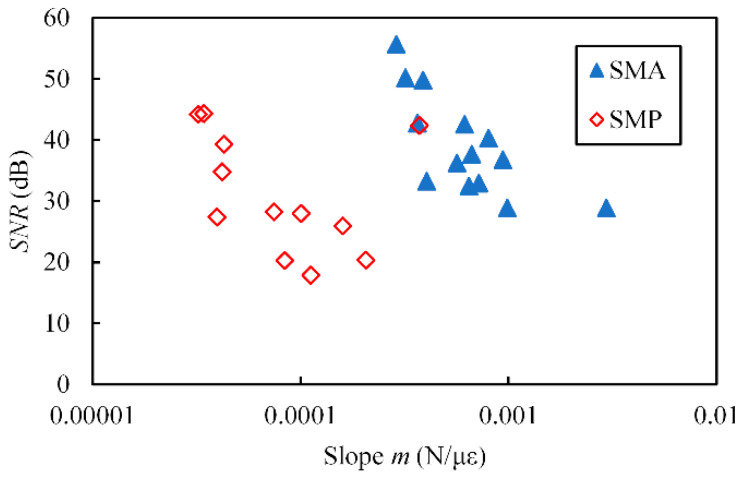
Relationship between slope *m* and *SNR*.

The relationship between *m* and *SNR* is shown in Figure 15. When the sensitivity of the sensor increased (namely, *m* and the measurement range decreased), the *SNR* also increased, which is an advantage of the proposed sensor. This tendency is different from that for a conventional FS [8].

As shown in this section, the proposed sensor has a simple structure and the method used to change the MRS is simple (based on only bending). Therefore, the sensor can be inexpensively implemented. In this study, it was challenging to change the bending position arbitrarily because we bent the sensors manually. Sun et al. developed an FS with a continuously adjustable resolution that uses a motor to change stiffness [4]. Based on a similar idea, to achieve continuous and accurate adjustment for our sensor, a jig that includes a mechanism to adjust the bending position can be used, as shown in Figure 14.

## 5. Conclusions

In this study, we proposed a variable-sensitivity FS that uses structural modification. The use of SMMs allows the sensor structure to be easily changed and fixed by controlling the temperature. We designed two sensors, one based on SMP and one based on SMA, and evaluated their prototypes experimentally. We bent and fixed the SMA and SMP plates at 90° in order to change the distance between the force application point and the detection area. The relationship between the applied force and the strain could be changed by bending the prototype sensor, which is consistent with the theoretical results. However, in some directions, there was a large hysteresis. One reason for the increase in hysteresis could be that the sensor tip moved on the indenter surface. Moreover, the tip of the sensor buckled and deformed. There was a difference between the experimental and theoretical values, which could be attributed to manufacturing errors. For example, since we manufactured and bent the sensors manually, the width and thickness were not constant, and the bending angle of the SMM plate was not exactly 90°. In a future study, we will design a jig to bend and heat the sensor to minimize these errors. Moreover, we will evaluate various sensor shapes (e.g., various cross-sectional shapes) under various experimental conditions (e.g., deformation speeds).

## Figures and Tables

**Table 1:** Dimensions of prototype SMA FS (average ± standard deviation from ten measurements).

	Sample No. 1	Sample No. 2	Sample No. 3
Length, *L* (mm)	84.4 ± 0.0	86.4 ± 0.1	89.5 ± 0.0
Width, *b* (mm)	5.2 ± 0.0	5.3 ± 0.0	5.2 ± 0.0
Thickness, *h* (mm)	0.69 ± 0.00	0.70 ± 0.00	0.70 ± 0.00
*x*_1_ (mm)	34.8 ± 0.4, 44.8 ± 0.4	38.5 ± 0.9, 42.6 ± 0.4, 47.8 ± 0.3	50.5 ± 0.4, 51.2 ± 0.3
*x*_2_ (mm)	32.8 ± 0.2, 23.8 ± 0.3	28.7 ± 0.4, 22.2 ± 0.3, 21.1 ± 0.3	13.7 ± 0.2, 18.9 ± 0.5

**Table 2:** Dimensions of prototype SMP FS (average ± standard deviation from ten measurements).

Width, *b* (mm)	6.8 ± 0.0
Thickness, *h* (mm)	0.82 ± 0.01
*x*_1_ (mm)	52.0 ± 0.4, 50.6 ± 0.6, 46.4 ± 0.5, 45.2 ± 0.2, 41.0 ± 0.6, 31.3 ± 0.2
*x*_2_ (mm)	13.3 ± 0.3, 12.5 ± 0.3, 16.9 ± 0.4, 17.8 ± 0.2, 21.6 ± 0.3, 31.5 ± 0.3

## Data Availability

Not applicable.
